# Electronic medical record implementation in tertiary care: factors influencing adoption of an electronic medical record in a cancer centre

**DOI:** 10.1186/s12913-020-06015-6

**Published:** 2021-01-06

**Authors:** Anna Janssen, Candice Donnelly, Elisabeth Elder, Nirmala Pathmanathan, Tim Shaw

**Affiliations:** 1 Faculty of Medicine and Health, Charles Perkins Centre, The University of Sydney, Level 2, Charles Perkins Centre D17, Sydney, NSW Australia; 2 Sydney West Translational Cancer Research Centre, Westmead Hospital, Sydney, NSW Australia; 3 Westmead Breast Cancer Institute, Western Sydney Local Health District, Sydney, NSW Australia

**Keywords:** Multidisciplinary care, Clinical informatics, Electronic medical records, Implementation, Digital health, Data, Technology

## Abstract

**Background:**

Electronic Medical Records (EMRs) are one of a range of digital health solutions that are key enablers of the data revolution transforming the health sector. They offer a wide range of benefits to health professionals, patients, researchers and other key stakeholders. However, effective implementation has proved challenging.

**Methods:**

A qualitative methodology was used in the study. Interviews were conducted with 12 clinical and administrative staff of a cancer centre at one-month pre-launch and eight clinical and administrative staff at 12-months post-launch of an EMR. Data from the interviews was collected via audio recording. Audio recordings were transcribed, de-identified and analysed to identify staff experiences with the EMR.

**Results:**

Data from the pre-implementation interviews were grouped into four categories: 1) Awareness and understanding of EMR; 2) Engagement in launch process; 3) Standardisation and completeness of data; 4) Effect on workload. Data from the post-launch interviews were grouped into six categories: 1) Standardisation and completeness of data; 2) Effect on workload; 3) Feature completeness and functionality; 4) Interaction with technical support; 5) Learning curve; 6) Buy-in from staff. Two categories: Standardisation and completeness of data and effect on workload were common across pre and post-implementation interviews.

**Conclusion:**

Findings from this study contribute new knowledge on barriers and enablers to the implementation of EMRs in complex clinical settings. Barriers to successful implementation include lack of technical support once the EMR has launched, health professional perception the EMR increases workload, and the learning curve for staff adequately familiarize themselves with using the EMR.

**Supplementary Information:**

The online version contains supplementary material available at 10.1186/s12913-020-06015-6.

## Background

Digital technologies are being widely adopted in the healthcare, leading to a widespread transformation of the sector. This transformation has included an increase in the personalisation of health care [1, 2] and potential for new approaches to facilitate research quality improvement initiatives and patient driven care [3]. Lately, digital technologies have been adopted by health sectors often for its better functioning and Electronic Medical Records (EMRs). EMRs are computerized information systems which collect, store and display patients’ information and typically replace paper-based medical records [4].

EMRs are key enablers of the data revolution transforming the health sector [5]. They incorporate a wide range of functionalities but can be broadly described as systems for presenting health, clinical or medical records in a digitised format [6]. The literature has acknowledged a number of benefits of using EMRs for healthcare professionals, patients, organisations and other stakeholders. Benefits include improving the quality of care for patients [6], improving timely access to data, and to potentially facilitate knowledge exchange for collaboration in multidisciplinary teams (MDTs) [7].

In spite of the wide range of potential benefits, physicians and other healthcare staffs can harness these supports only if EMRs are implemented [4]. However, it has been noted in the literature that there is a notable gap in understanding about the best ways to implement digital health [8, 9]. The implementation of EMRs is particularly complex and requires a range of technical, human and organisational factors to be considered [7]. A comprehensive review of the literature identified eight categories of barriers to EMR adoption: Financial, Technical, Time, Psychological, Social, Legal, Organizational and Change process [4]. Financial, time/workload and technical barriers are the most frequently cited challenges in the literature [7].

Although the literature has identified some of the barriers and enablers to implementing EMRs in healthcare, much of this research has focused on primary care [10]. There is currently a dearth of literature on the implementation of EMRs in cancer care, which is a unique setting for implementing technology. The gold standard for the delivery of cancer care is the use of MDTs. MDTs describe a group of health professionals from a range of specialties who work together on a regular basis to deliver evidence-based care to patients [11]. Successful implementation of EMRs in a multidisciplinary setting requires consideration of the unique challenges to implementing EMRs for health professionals across a range of specialties, and for administrative staff.

This study described in this manuscript aimed to explore the barriers and enablers to implementing an EMR in a metropolitan tertiary cancer service.

## Methods

### Study design

A qualitative methodology was used to collect data on the experiences of clinical and administrative staff at pre- and post- launch of a new EMR.

### Participants and study setting

The EMR was implemented in November 2017 in a breast cancer service within Western Sydney Local Health District. The cancer service is located across three hospitals within Western Sydney Local Health District (LHD). The main centre is co-located with a screening clinic and registers approximately 500 new breast cancer patients per year. Staff at the cancer centre had used another EMR platform for 10 years prior to the launch of the new EMR investigated in this study. The existing platform was a bespoke SQL database that no longer met the needs of the service. This EMR was predominantly used by administrative staff who entered data from paper records completed by clinical staff as well as clinicians doing follow-up with patients, had restricted interoperability with the main hospital EMR (Cerner) and was not used widely across the various disciplines within the service. The new EMR was developed in a Cerner Millennium platform with bespoke built multidisciplinary breast cancer fields for data entry at point-of-care. The development was a collaboration between key clinical representatives of the breast cancer centre and the LHD Information Communication and Technology service over a period of several years.

All clinical and administrative staff within the service were eligible to participate in the study, but due to the size of the center not all staff opted to participate. A purposeful sample were recruited to participate in 30–60 min phone interviews about their experiences with the EMR. A total of 12 participants consented to participate in the pre-launch interviews, three in administrative or research roles and nine in clinical roles. A total of eight participants consented to participate in the post-launch interviews, two in administrative or research roles and six in clinical roles. Participants were from a range of disciplines including medical oncology, radiation oncology, surgery, pathology, nursing and clinical administration.

### Procedures

Potential participants were recruited via email to participate in the study. Consent was provided either through return of a signed consent form, or via confirmation in the email response. At the start of each interviewee consent to participate in the interview and consent to record the interview was once again confirmed.

Interviews were conducted with staff within the cancer centre one-month pre-launch and twelve months post-launch of the EMR. Potential participants for the pre-implementation interviews were identified by members of the Advisory Committee that oversee the development of data improvement projects within the organisation. A member of the research team contacted all eligible participants via email and scheduled a pre-launch interview. All participants who completed a pre-launch interview were invited to participate in an interview post-launch.

Both pre and post launch interviews were conducted face to face or via phone at a time convenient to the participant. Interviews took between 30 and 60 min and were conducted by a researcher experienced in qualitative methods. Interviews were audio recorded. Audio recordings were transcribed by a commercial transcription service, prior to de-identification by one member of the research team.

*Refer to* Additional file 1*to see the interview guide.*

### Analysis

Analysis was conducted to identify key themes and subthemes. These themes were then refined to reduce redundancy and emphasise prominent groupings. During analysis, illustrative quotes were identified and grouped them by themes and sub-themes.

### Ethics

The study was granted ethical approval by the Western Sydney Local Health District Human Research Ethics Committee’s Executive Committee (protocol/approval number:4678). All participants provided written consent to participate in the study by return of an email, verbal consent was confirmed at the start of each interview.

## Results

### Pre-implementation interview findings

#### Pre-implementation interview findings were grouped into 4 categories

- Awareness and understanding of electronic medical record
- Engagement in launch process
- Effect on workload
- Standardisation and completeness of data

Refer to Table 1for exemplar quotes related to these themes.

**Table 1:** Pre-implementation semi-structured interview illustrative quotes categorized by theme

Category	Quote	Participant
Awareness and understanding of electronic medical record	“Everything will go in it. So, basically, instead of writing in the notes and ticking boxes I’m going to be clicking through boxes on a computer. That’s the way I understand it.”	1.2
	“I think the old record will sort of phase out and new record will basically supersede that and also the paper record will be gone, so the EMR will just replace the paper record. That’s my understanding, yeah”	1.4
	“it is going to be an Electronic Medical Record that will be used for patient care and also simultaneously generate data that can be used for quality improvement and research.”	1.6
	“I’m hoping that it would be a more complete dataset. I don’t have to wait for admin staff to complete the data. It will be more prospectively data collection, because clinicians, I think it’s my understanding that when EMR will be up running, clinicians will be doing lots of data entry, that’s the expectation.”	1.1
	“I think it will mean that I will have a better understanding about the patient when they’re on the phone, so that I can make valid and informed, give them informed information based on the correct history and diagnoses and pathology and doctors letters.”	1.11
Engagement in launch process	“I think timewise it’s good timing [launching at the end of the year] because it is getting closer to the slowdown period and maybe we’ll have a bit of more time to train ourselves and learn.”	1.12
	“There have been a number of opportunities for us to go to [to training], but unfortunately most of the times that have been in ... Most of the nursing EMR training has been on those days, at very difficult times. It’s been a bit stressful trying to get to them.”	1.11
	“there were a time everyone was involved [in EMR implementation] and everyone had the opportunity to look through the forms, because the forms were built on the basis of the [the existing platform]... Everyone had the chance to talk about it and discuss it.”	1.1
	“I had a few opportunities to work with them [the implementation team]. When my manager was on holiday they actually called me to have some kind of meeting and some input on how the admin workflow goes in the clinic...I didn’t know the exact workflow in the EMR so I didn’t have any much concern but I gave some feedback, inputs, and all.”	1.4
	“I had my training sessions this week with the EMR, and [the traininer] was saying to me that his impression, teaching people through this system has been generally very positive. So, people in general have a positive attitude towards it. At least at this point.”	1.5
	“We’ve done lots of testing, but you never know until you implement it and you’re using it in the clinic, then only will you come up with what else is wrong with it.”	1.8
	“To a degree, even today we’re in a training environment where some of the functions are not even enabled on the staff EMR. How can you go through and practice if you don’t even have it? I guess this was the period to find out that there were issues there, which is good before we go live. But again, until you real world, you can do a little mock whatever. It’s like getting married, you can go through all your practice routine, but on the day it’s a completely different story. I’m sitting back, I’ve reduced clinics, I’m anticipating the worst, hoping for the best.”	1.3
	“it’s pretty good and mean we all just get one opportunity for training. It would have been nice to have a couple. Yeah, moderately satisfied. But I was involved in the design so for me it’s a bit more fluent.”	1.8
Standardisation and completeness of data	“the accuracy and quality of data will be greater as a result because it should hopefully there will be less transcription error... I think it’ll [the EMR], hopefully, it’ll increase integrity and the rigorousness of the data.”	1.2
	“The only one of the big positives I mentioned before is obviously everything that’s contained in that one record, and we don’t have to search for a file.”	1.3
	“I mean hopefully all the relevant information are scanned in on time so that you can actually just, it doesn’t matter where you are, you’d be able to access that, so that will be useful...You can access it wherever you are, I think that’s, rather than physically going find a file. I think that will be the biggest thing.”	1.5
	“I think it’s going to improve a lot in terms of finding information easily, because there’s a lot of time spent, particularly, for the admin people. But also, if you ask for a file, somebody has to actually find the file, pull it out, then looking into it, and a lot of various ... When we have the new system up and running, we’ll be able to just log on and look up all the information electronically.”	1.6
	“hopefully, we will find that the data that is collected is very accurate. I’m also hoping that it will be complete data. All of that, of course, depends on how much, how diligent people are in filling out the digital forms. But because they are also going to be used for clinical purposes and for correspondence with clinicians, so unless you actually enter information into the system, there won’t be anything coming out the other end. And I think that is going to be a safeguard for the data to be completely entered.”	1.6
	“It will minimise the data entry errors ... also all the clinical data will be in there. A patient summary will be generated from the clinical data and that will be very useful for us to access for clinical purposes to get to know about the past history of the patient and also for research purposes it will make a huge difference that we’ll have access to all of this data.”	1.8
Effect on workload	“I think it’ll just be a bit slower initially, more than anything. But who knows, it might work really brilliantly, and save a bit of time in the end. I’m optimistic, put it that way.”	1.11
	I’m pretty sure it will be a bit of a challenge but you can’t achieve any good things without a challenge. I think the first couple of weeks to start will be challenging, but after that you spend more time initially but save along the years.	1.12
	“I’m really looking forward to doing EMR because I think positively, it may be a good change for us... our expectation is that maybe the workload will be little bit less. That stress will be less because now we are struggling with short staff and stuff like that. It’s hard to meet up with the deadlines and we push ourselves to complete that task, it’s sometimes really hard so we are hoping to get some relief off that.”	1.4

##### Awareness and understanding of electronic medical record

All participants were aware of the upcoming EMR launch within the service. The majority of participants were enthusiastic about the potential of the EMR, but many acknowledged uncertainty in what the additional capabilities and functionalities of the new EMR would be.

The participants who held the greatest knowledge of the functionality of the new EMR had been involved in the design and implementation of the EMR. Outside of this group, almost all participants indicated a basic understanding that the EMR would replace the current process of using paper records at point of care with digitised data entry into the EMR. It was also widely understood that the new EMR would change the data entry process to be completed by clinical staff rather than administrative staff. Some participants also indicated that the new EMR would replace the existing database used to store clinical notes, anticipating that this would be useful for research and quality improvement activities.

Finally, a number of participants discussed the use of the new EMR in MDT meetings instead of the existing process where administrative staff manually extract and collate relevant patient information for discussion. Most of the participants who understood this application of the EMR were clinical staff. Most participants thought the use of the EMR in MDT meetings would be beneficial, particularly for early career clinicians who would be able to learn from the process of using the record in meetings.

##### Engagement in launch process

All participants indicated that they had been engaged on some level during the implementation process for the new EMR. Most participants had been involved in training sessions in the weeks prior to the EMR launch, or had been provided an opportunity to participate even if they hadn’t attended. A minority of participants indicated they had an active role in the design of the new EMR in the 18-month development period.

Although participants were happy with the level of engagement they had with the development and implementation of the EMR, the majority weren’t sure whether their needs had been captured in the final EMR design. A minority of participants indicated that features they had requested were not being made available in the initial launch of the EMR, but anticipated the inclusion of those features in the future. Other participants felt their requests for particular EMR features had been included, but were not sure how what they had requested would function once the EMR was made available to them. This was because request for features were often made prior to using the EMR or when there had only been limited use of the EMR. It was anticipated that there would be a need to refine the design once the EMR had launched in order to align it with the needs of the service.

##### Standardisation and completeness of data

Participants perceived the EMR to be beneficial for improving the quality of clinical data collected within their centre in the future. This was one of the most consistently stated benefits of implementing a new EMR. The anticipated benefits of the improved data collection and quality varied across participants, but included a reduction in incomplete data entry for patient records due to mandatory fields, a more holistic view of the patient record and opportunities to access data for research purposes.

A minority of participants raised concerns about the EMR’s effect on standardisation and data completeness. Some participants raised concerns that the EMR was not integrated with other clinical data repositories within the local health district (outside of the NSW Health EMR) and that interoperability would continue to be an issue. Another participant raised concern about the limitations of the EMR to capture data that was appropriate for their discipline specialty.

##### Effect on workload

The majority of participants discussed the perceived effect of the EMR on their workload. Generally, participants were concerned that the EMR may negatively impact workload, particularly for clinical staff who would be required to complete data entry tasks. Although workload concerns were raised frequently, multiple participants also acknowledged that this issue was likely to resolve after launch when both clinical and administrative staff became more familiar with the technology and integrated it into their routine.

Although most participants felt the EMR would increase workload, a minority of participants felt that the EMR implementation could decrease their workload. Currently, a number of participants described having to manage and search through a large number of paper files to access information. Participants anticipated that being able to access information digitally would be more efficient. Other participants felt it would be beneficial to be able to access data in the EMR remotely allowing more flexibility over where work was undertaken.

### Post-launch interview findings

#### Post-implementation interview findings were grouped into 5 categories

- Standardisation and completeness of data
- Effect on workload
- Feature completeness and functionality
- Learning curve
- Buy-in from staff

Refer to Table 2for exemplar quotes related to these themes.

**Table 2:** Post-implementation semi-structured interview illustrative quotes categorized by theme

Category	Quote	Participant
Standardisation and completeness of data	“One thing I like, now we are all in the same picture. Doctors, nurses and admin staff, everyone.”	2.1
	“I think it is actually the generation of documentations: the letters, the fact that the template can be pulled across as a letter, I think is really useful. The template that we use now is a lot easier to use than it was.”	2.2
	“it just minimises the risk of there being errors, because everything is done straight away.”	2.2
Effect on workload	“I think it’s a lot more efficient, it cuts out a lot of excessive work, for us at least. And it’s a lot more accessible, the information.”	2.2
	“I’m really enjoying the system … it saves a lot of time.”	2.2
	“at first I thought it is going to be very easy, but now looking at the EMR, we’ve got a little bit more to do at this stage, because I think that a lot, little things need to be fixed there”	2.1
Feature completeness and functionality	“if we open up the wrong encounter, we might generate a letter in the wrong encounter. That’s something that happens fairly commonly, or if we forget to add a patient to a patient list at the end of it all, then sometimes the admin staff get a bit cranky about that. The actual system itself, I think is really good.”	2.2
	“issues with the features will lead to lack of usage and data loss”	2.3
	“we actually phoned the EMR team and they said they are going to fix it, but not yet.”	2.1
	“So, we have ... she’s not actually dedicated support, but she is someone who’s got excellent knowledge of Cerner now. And she’s sort of very responsive to any of our requests. So, she’s been really good, but prior to that ... this has been in the last, I would say, 12 months, but prior to that, we didn’t’ have a lot of support at all. So, we just had to kind of fumble our way through it.”	2.4
Learning Curve	“everyone always finds it a bit of a learning curve...but after a week or two everyone picks it up very quickly. And then it becomes very easy to do.”	2.2
Buy-in from staff	“they can’t see the benefit of using it still. I know that there’s the issues with their formatting, but they’ve been very slow to try and maybe get motivated to use it or try and resolve the issue. So, that’s all been from our end, pushing, pushing, pushing, trying to get them onboard with it. So, I think, once people start using it, then you start to see the benefits of using it and how quick it actually can make things quite efficient.”	2.4

A total of eight interviews were conducted with staff within the cancer center. Of these interviews, three were undertaken with administrative staff and five were undertaken with clinical staff. Clinical staff represented a range of specialties including radiation oncology, surgery and pathology.

Data analysed from the interviews was classified into six categories: 1) Standardisation of documentation and completeness of data; 2) Effect on workload; 3) Feature completeness and functionality; 4) Interaction with technical support; 5) Learning curve; 6) Buy-in from staff.

##### Standardisation and completeness of data

Both administrative and clinical staff indicated benefits of interoperability with the main hospital EMR and centre-wide access to the same information in the EMR. This was valuable when patients had been transferred to the centre from other hospital departments as patient information was easily accessible, enabling more efficient internal transfer for patients. It was also noted as a useful reference for finding initial consult information for returning patients that the clinical staff had not seen for a long time.

The EMR was noted as valuable for improving completeness of data on MDT recommendations. The new process of live data entry during MDT meetings allows all members to ensure the accuracy of the data in real-time, thus reducing recall bias that may have occurred when data was entered from notes after the MDT meeting. The centre had also installed three large LCD screens in the MDT meeting room to easily visualise patient information from multiple systems at the same time as viewing the EMR. One participant noted that data entry during the MDT meeting led to increased recording of longer-term recommendations for patients.

##### Effect on workload

The EMR was perceived to have both positive and negative impacts on workload. A participant reported that the EMR increased efficiency by making information more accessible. Once familiar with the platform, it was described as relatively easy to navigate the EMR and input patient data. With regards to follow-up patients, data entry was noted as particularly efficient, only taking a few minutes, though it was more time consuming for new patients. Other benefits included decreased time in finding and reviewing information prior to a patient consultation, when compared with the previous process of manually searching through paper records.

Participants noted increased efficiency and reduced risk of errors in the new process of clinical staff entering data directly into the EMR rather than the previous process of administrative staff transcribing paper records. One participant noted the increased accountability for clinical staff to input data into the EMR. However, concerns were raised that some senior clinicians had experienced an increased workload as they complete EMR data entry themselves, due to uncertainty regarding whether early career doctors were inputting data consistently.

Although some participants felt the EMR was more efficient, others felt it increased workload. Concern was also raised about the unexpected issues when the new EMR did not allow staff to complete simple tasks that were previously automated, including the inability to bulk-print letters. The EMR was described as having increased workload for administrative staff due to increased time spent on auditing and completing the letters automatically generated from the EMR, following-up clinical staff for completion of data needed to generate the letters and repetitively scanning documents in to the EMR.

##### Feature completeness and functionality

A number of participants raised concerns about issues with features, or functionality of features in the EMR, and ranged in degree of severity, from usability to issues effecting data quality and workload. Some of these issues included not having drop down menus to select a doctor or provider name in the EMR or generating letters from the incorrect clinical encounter. Letter generation was a largely cited concern. Participants noted that the process of letter generation was cumbersome and often the letter contained the incorrect content. Furthermore, the letter layout was described as unprofessional by some interviewees as the template put constraints on the content and structure on the letter, preventing clinicians to generate their patient letters in a preferred style.

A significant concern, was the lack of the patient summary page which provided a single-page overview of the patients treatment and other clinical interactions in the EMR. The inclusion of a patient summary view was an important feature of the new EMRs design, and its sub-optimal implementation was a perceived barrier to use of the new EMR. This feature was intended for quick and easy access to pertinent patient information in one instance. The patient summary was also intended to be used for MDT meetings. Participants noted that due to a lack of the ‘patient summary’ page, the required navigation across tabs to retrieve all the information needed was time consuming.

Another major functionality issue was lack of auto completion of fields in the EMR. Concerns were raised about GP information not auto-populating as it did in the previous system. As this functionality was also not successfully executed, an unexpected technical issue that occurred was that patient information did not transfer across clinical encounters. There were some issues that occurred in the first 3 months of implementation when forms were not auto-populating and there was a loss of data. Finally, some participants noted that not all forms suited the type of appointment e.g. second appointment for surgical decision, making it difficult to know where the data should be entered appropriately. Another highlighted issue was the lack of flexibility in the EMR drop-down menus or pre-set tick box options. One clinical staff member noted this was particularly concerning as these options should incorporate emerging evidence, such as new drugs.

##### Learning curve

There were some concerns about the difficulty in remembering how to enter data into the new EMR fields, particularly for staff that didn’t interact with it regularly. The platform was different to that found in other clinical settings which also meant new staff had a learning curve to familiarise themselves with how to use the system. However, it was noted that it had typically taken one to 2 weeks to overcome the learning curve. After this time, the EMR usability was reported positively. One participant noted that the EMR may be challenging to learn to use for relief staff who may only use it for one or 2 weeks.

##### Buy-in from staff

Some participants raised concerns about lack of buy-in to the EMR across clinical disciplines that work within and in collaboration within the centre, which led to gaps in available information. There were also concerns that some people had ceased using the EMR because of issues with the data fields. These issues included drop down menus in the EMR not including treatment options the clinicians wanted to use, and other issues relating to navigating the EMR.

## Discussion

This study presents findings describing the implementation of EMR in a cancer care centre. The findings are broadly aligned with the literature on barriers to implementation of EMRs, particularly in regard to the central role of Information Technology (IT) support during the post-implementation period [2, 7]. However, findings from this study suggested that staff were prepared to overlook challenges in functionality or technical support due to a perception the system would become beneficial over time as system errors would be addressed.

Findings from this study showed that prior to launch, study participants were generally enthusiastic about the EMR and optimistic it would improve processes. A core component of this enthusiasm was the perceived engagement during the pre-implementation process. A number of participants were provided an opportunity to be involved in the design of the EMR, and all participants noted they had an opportunity to participate in EMR training sessions. Whilst research has showed the value of involving health professionals in EMR to improve uptake [12], there is little research describing the important role of delivering flexible training to staff on the use of the new system in the lead up to implementation. The value of using education and training as a tool in successful implementation has been acknowledged in the literature [12], with findings from this study showing it is as important when implementing digital technologies as in other areas.

The literature has shown that clinician resistance is a major barrier to EMR implementation as they are the largest user group of EMR systems [4]. However, findings from this study identified some perceived benefits that have not previously been recognized in the literature. The EMR was particularly well received by early career doctors who had found it easier for documentation, time saving, effective for improving record completeness and beneficial for guiding clinical consultations in highlighting what should be recorded. Clinical staff also felt the EMR enabled senior specialists to act in a more traditional consultant role, with early career doctors completing data entry. By incorporating the EMR into MDT meetings there was a perceived benefit to early career doctors as they had an opportunity to engage more actively with patient records and ask questions about treatment decisions. The central role of informal education opportunities in the training and mentorship of early career doctors has been acknowledged in the literature [13, 14]. Furthermore, there literature on health professions education has acknowledged the untapped potential of EMR data and other clinical data sets for supporting high-quality learning environments [15]. Findings from this study suggest placing an emphasis on these benefits when implementing digital technologies to enhance EMR uptake.

Although findings from this study identified benefits of the EMR, there were also a number of challenges identified which acted as barriers to engagement with the new system. These challenges included feature completeness and reliability, as well as poor communication from IT regarding feasibility of achieving required program features and in addressing EMR issues once implemented. Interestingly, the major challenges implementing the EMR were external to the control of the clinical team implementing the system. The literature has noted that lack of infrastructure and technical supports is a major barrier preventing the harnessing of EMR data by the staff [16], and for making health data actionable more broadly [15]. Findings from this study emphasise the enormity of overcoming resourcing and infrastructure challenges if digital technologies are to be effectively implemented and sustained in the health sector. Finally, the challenges faced when successfully implementing an EMR in this study highlight the need for technical and clinical experts to collaborate closely from the outset when developing digital technologies for healthcare, in order for those technologies to capture the complexity of clinical care.

A limitation of this study is that, although all staff at the study site were invited to participate, not all chose to. As a result the perspectives of these staff members might not have been captured in this study. Further, not all participants who participated in pre-implementation interviews chose to participate in post-implementation interviews. As such some of the perspectives of interviewees pre-implementation are not captured in the post-implementation findings.

## Conclusion

Digital technologies such as EMRs have great potential to improve the quality, equity and cost of healthcare. However, the health sector is still struggling to implement these technologies in a way that leads to sustained use. Barriers to successful implementation of EMRs include lack of technical support, perceived increase in workload and a learning curve to adequately familiarise with the feature set of the EMR. Enablers of successful implementation include clinician engagement in the design and roll-out of the EMR, and training and upskilling of all end-users of the EMR. Although there may be challenges in the usability of EMRs right after its implementation, staff will be encouraged to use it if they perceive improved features of EMR are imminent and has potential benefit to patient care and workflow. However, it is important that continuous supports are provided to ensure buy-in from health professional is not lost.

## Supplementary Information

**Additional file 1.**

## Data Availability

The datasets used and/or analysed during the current study are available from the corresponding author on reasonable request.

## Abbreviation

EMR: Electronic Medical Record/s
