# Supplementary material for: Electronic medical record implementation in tertiary care: factors influencing adoption of an electronic medical record in a cancer centre
Source: BMC Health Serv Res. 2021 Jan 6;21:23. doi: 10.1186/s12913-020-06015-6 (PMC7789279; doi:10.1186/s12913-020-06015-6)
Supplement: Supplementary file 1 — Additional file 1. [file 12913_2020_6015_MOESM1_ESM.pdf]

INTERVIEW GUIDE

Pre eMR Implementation:

1. What is your understanding of what the new eMR will be like (ie: what sort of data will go in to it, what systems might it replace)?
2. How do you currently interact with the clinical databases in the hospital?
3. How do you anticipate you will in interact with the eMR when it is implemented?
4. Is there anything about the eMR you are particularly excited about?
5. Is there anything about the eMR you think might be challenging to adapt to?
6. To what extent do you think the eMR will have any impact on the quality of clinical data collected in the hospital?
7. To what extent do you feel the eMR will improve your ability to access clinical data collected in the hospital?
8. To what extent do you feel the eMR team has engaged you in the implementation process?
9. Do you have any general comments about the eMR you would like to make?

Post eMR Implementation follow up:

1. How have you found the new eMR?
2. Is there anything you have really liked about the eMR?
3. Is there anything that has been challenging about the eMR?
4. How supportive have the development team been in addressing any concerns you have had about the eMR?
5. Have you had any feedback from colleagues about the eMR?
6. Has anything surprised you about the eMR?
7. Do you have any general comments about the App so far?
